# Neutralizing IFN-**γ** autoantibodies are rare and pathogenic in HLA-DRB1*15:02 or 16:02 individuals

**DOI:** 10.1172/JCI178263

**Published:** 2024-03-12

**Authors:** Jessica N. Peel, Rui Yang, Tom Le Voyer, Adrian Gervais, Jérémie Rosain, Paul Bastard, Anish Behere, Axel Cederholm, Aaron Bodansky, Yoann Seeleuthner, Clément Conil, Jing-Ya Ding, Wei-Te Lei, Lucy Bizien, Camille Soudee, Mélanie Migaud, Masato Ogishi, Ahmad Yatim, Danyel Lee, Jonathan Bohlen, Thomas Perpoint, Laura Perez, Fernando Messina, Roxana Genet, Ludovic Karkowski, Mathieu Blot, Emmanuel Lafont, Laurie Toullec, Claire Goulvestre, Souad Mehlal-Sedkaoui, Jérôme Sallette, Fernando Martin, Anne Puel, Emmanuelle Jouanguy, CONSTANCES cohort, 3C-Dijon Study, Etablissement du Sang study group, Mark S. Anderson, Nils Landegren, Pierre Tiberghien, Laurent Abel, Stéphanie Boisson-Dupuis, Jacinta Bustamante, Cheng-Lung Ku, Jean-Laurent Casanova

**Affiliations:** 1 St. Giles Laboratory of Human Genetics of Infectious Diseases, The Rockefeller University, New York, New York, USA.; 2 Laboratory of Human Genetics of Infectious Diseases, Inserm U1163, Paris, France.; 3 Paris Cité University, Imagine Institute, Paris, France.; 4 Clinical Immunology Department, Assistance Publique Hôpitaux de Paris (AP-HP), Saint-Louis Hospital, Paris, France.; 5 Study Center for Primary Immunodeficiencies and; 6 Pediatric Hematology-Immunology and Rheumatology Unit, Necker Hospital for Sick Children, Assistante Publique-Hôpitaux de Paris (AP-HP), Paris, France.; 7 Science for Life Laboratory, Department of Medical Biochemistry and Microbiology, Uppsala University, Uppsala, Sweden.; 8 Department of Pediatric Critical Care Medicine and; 9 Department of Medicine, UCSF, San Francisco, California, USA.; 10 Laboratory of Human Immunology and Infectious Disease, Graduate Institute of Clinical Medical Sciences; Center for Molecular and Clinical Immunology, Chang Gung University, Taoyuan, Taiwan.; 11 Department of Pediatrics, Hsinchu Municipal MacKay Children’s Hospital, Hsinchu, Taiwan.; 12 Infectious and Tropical Diseases Service, Hospices Civils of Lyon, Lyon, France.; 13 Immunology and Rheumatology Unit, Prof. Dr. Juan P. Garrahan National Hospital of Pediatrics, Buenos Aires, Argentina.; 14 Mycology Unit, Dr. Francisco J. Muñiz Hospital, Buenos Aires, Argentina.; 15 Infectious Diseases Service, Regional Hospital of Metz-Thionville, France.; 16 Deparement of Internal Medicine, Sainte Anne Armed Forces Teaching Hospital, Toulon, France.; 17 Department of Infectious Diseases, Dijon-Bourgogne University Hospital, Dijon, France.; 18 Department of Infectious Diseases and Tropical Medicine, Paris Cité University, Necker Hospital for Sick Children and; 19 Laboratory of Immunology, Cochin hospital, AP-HP, Paris, France.; 20 Cerba, Saint-Ouen l’Aumône, France.; 21 Cerba HealthCare, Issy-les-Moulineaux, France.; 22 Details are available in the Supplemental Acknowledgments.; 23 Centre for Molecular Medicine, Department of Medicine (Solna), Karolinska Institute, Stockholm, Sweden.; 24 Etablissement Français Du Sang, La Plaine Saint-Denis, France.; 25 20UMR1098 RIGHT, INSERM, EFS, Université de Franche-Comté, Besançon, France.; 26 Department of Nephrology, Chang Gung Memorial Hospital, Taoyuan, Taiwan.; 27 Howard Hughes Medical Institute, New York, New York, USA.

**Keywords:** Genetics, Immunology, Autoimmune diseases, Cytokines

## Abstract

**BACKGROUND:**

Weakly virulent environmental mycobacteria (EM) can cause severe disease in HLA-DRB1*15:02 or 16:02 adults harboring neutralizing anti-IFN-γ autoantibodies (nAIGAs). The overall prevalence of nAIGAs in the general population is unknown, as are the penetrance of nAIGAs in HLA-DRB1*15:02 or 16:02 individuals and the proportion of patients with unexplained, adult-onset EM infections carrying nAIGAs.

**METHODS:**

This study analyzed the detection and neutralization of anti-IFN-γ autoantibodies (auto-Abs) from 8,430 healthy individuals of the general population, 257 HLA-DRB1*15:02 or 16:02 carriers, 1,063 patients with autoimmune disease, and 497 patients with unexplained severe disease due to EM.

**RESULTS:**

We found that anti-IFN-γ auto-Abs detected in 4,148 of 8,430 healthy individuals (49.2%) from the general population of an unknown HLA-DRB1 genotype were not neutralizing. Moreover, we did not find nAIGAs in 257 individuals carrying HLA-DRB1* 15:02 or 16:02. Additionally, nAIGAs were absent in 1,063 patients with an autoimmune disease. Finally, 7 of 497 patients (1.4%) with unexplained severe disease due to EM harbored nAIGAs.

**CONCLUSION:**

These findings suggest that nAIGAs are isolated and that their penetrance in HLA-DRB1*15:02 or 16:02 individuals is low, implying that they may be triggered by rare germline or somatic variants. In contrast, the risk of mycobacterial disease in patients with nAIGAs is high, confirming that these nAIGAs are the cause of EM disease.

**FUNDING:**

The Laboratory of Human Genetics of Infectious Diseases is supported by the Howard Hughes Medical Institute, the Rockefeller University, the St. Giles Foundation, the National Institutes of Health (NIH) (R01AI095983 and U19AIN1625568), the National Center for Advancing Translational Sciences (NCATS), the NIH Clinical and Translational Science Award (CTSA) program (UL1 TR001866), the French National Research Agency (ANR) under the “Investments for the Future” program (ANR-10-IAHU-01), the Integrative Biology of Emerging Infectious Diseases Laboratory of Excellence (ANR-10-LABX-62-IBEID), ANR-GENMSMD (ANR-16-CE17-0005-01), ANR-MAFMACRO (ANR-22-CE92-0008), ANRSECTZ170784, the French Foundation for Medical Research (FRM) (EQU201903007798), the ANRS-COV05, ANR GENVIR (ANR-20-CE93-003), and ANR AI2D (ANR-22-CE15-0046) projects, the ANR-RHU program (ANR-21-RHUS-08-COVIFERON), the European Union’s Horizon 2020 research and innovation program under grant agreement no. 824110 (EASI-genomics), the Square Foundation, Grandir - Fonds de solidarité pour l’enfance, the Fondation du Souffle, the SCOR Corporate Foundation for Science, the Battersea & Bowery Advisory Group, William E. Ford, *General Atlantic’s* Chairman and Chief Executive Officer, Gabriel Caillaux, General Atlantic’s Co-President, Managing Director, and Head of business in EMEA, and the General Atlantic Foundation, Institut National de la Santé et de la Recherche Médicale (INSERM) and of Paris Cité University. JR was supported by the INSERM PhD program for doctors of pharmacy (poste d’accueil INSERM). JR and TLV were supported by the Bettencourt-Schueller Foundation and the MD-PhD program of the Imagine Institute. MO was supported by the David Rockefeller Graduate Program, the Funai Foundation for Information Technology (FFIT), the Honjo International Scholarship Foundation (HISF), and the New York Hideyo Noguchi Memorial Society (HNMS).

## Abstract 2

Individuals harboring anti-IFN-g autoantibodies are susceptible to infection with avirulent mycobacteria.

## Introduction

Mendelian susceptibility to mycobacterial disease (MSMD) is a rare inherited condition characterized by susceptibility to infection by weakly virulent mycobacteria, including BCG vaccines and environmental mycobacteria (EM) (1–4). The patients are also vulnerable to the more virulent *Mycobacterium tuberculosis* and other intramacrophagic bacteria, fungi, and parasites (5–7). MSMD is typically isolated, although it can be syndromic if strongly associated with other infectious or noninfectious clinical phenotypes (7). Currently, germline variants in 22 MSMD-causing genes are involved in the production of interferon-γ (IFN-γ) (*IFNG, IL12B, IL12RB1, IL12RB2, IL23R, ISG15, MCTS1, RORC, TBX21,* and *TYK2*), cellular responses to IFN-γ (*CYBB, JAK1, IFNGR1, IFNGR2, STAT1,* and *USP18*)*,* or both (*IRF1*, *IRF8, NEMO,* and *SPPL2A*) (5–11). CCR2 deficiency underlies MSMD through impaired monocyte recruitment to infected tissues (12). The mechanism of MSMD in patients with *ZNFX1* variants is unknown (13). Arising primarily in childhood, the severity and penetrance of MSMD vary considerably between genetic etiologies and are inversely correlated with residual IFN-γ activity (5–7, 9, 14). Therefore, IFN-γ is not only indispensable for host defense against mycobacteria; it is also a quantitative trait that determines the outcome of mycobacterial disease (15, 16).

Potently neutralizing anti-IFN-γ autoantibodies (nAIGAs) can underlie a rare, acquired, adult-onset IFN-γ deficiency, initially characterized by vulnerability to infection with EM (17–20). Thus, the production of nAIGAs results in an autoimmune phenocopy of MSMD (17, 18, 21, 22). Like MSMD, the clinical manifestations can be diverse, including infections caused by other intramacrophagic pathogens, such as *Talaromyces marneffei* (22–26). Comorbidities, such as endocrine disorders, autoimmune disease, and cancers have been reported in some patients (24, 27). However, in contrast to MSMD, of the 600 reported cases since 2003 most individuals were adults (median age 55 yrs, approximately 87.6% between 40–87 yrs) (20) and most originate from Southeast Asia (17, 18, 20–22, 26). Diverging across different ethnic populations, HLA haplotypes are among the strongest genetic factors associated with autoimmune disease (28). Remarkably, most patients with nAIGAs carry at least 1 allele of HLA-DRB1*15:02 or 16:02 (29, 30). To date, there is no other known genetic determinant of nAIGAs.

Despite their significance in the disruption of IFN-γ–mediated immunity, the prevalence of nAIGAs in the general population is unknown, as is the penetrance of the nAIGAs in HLA-DRB1*15:02 or 16:02 individuals, and the proportion of patients with unexplained, adult-onset EM infections carrying nAIGAs. Remarkably, approximately 4%–8% of the general population aged over 70 years harbor autoantibodies (auto-Abs) neutralizing IFN-α/ω (31), which can underlie severe COVID-19 pneumonia (31, 32), MERS critical pneumonia (33), influenza critical pneumonia (34), West Nile virus encephalitis (35), and adverse reactions to the live attenuated yellow fever vaccine (36). Moreover, the incidence of infection with EM is increased in the elderly (37). Finally, previous reports have suggested that auto-Abs to IFN-γ are commonly found in the general population (38). We therefore set out to determine the proportion of individuals carrying neutralizing and nonneutralizing auto-Abs to IFN-γ in the general population, including HLA-DRB1*15:02 or 16:02 individuals. We also aimed to estimate the proportion of individuals with EM disease who carry nAIGAs. Finally, we analyzed the autoimmune landscape of these patients.

## Results

### Auto-Abs against IFN-γ are common in the general population.

**Figure 1:**
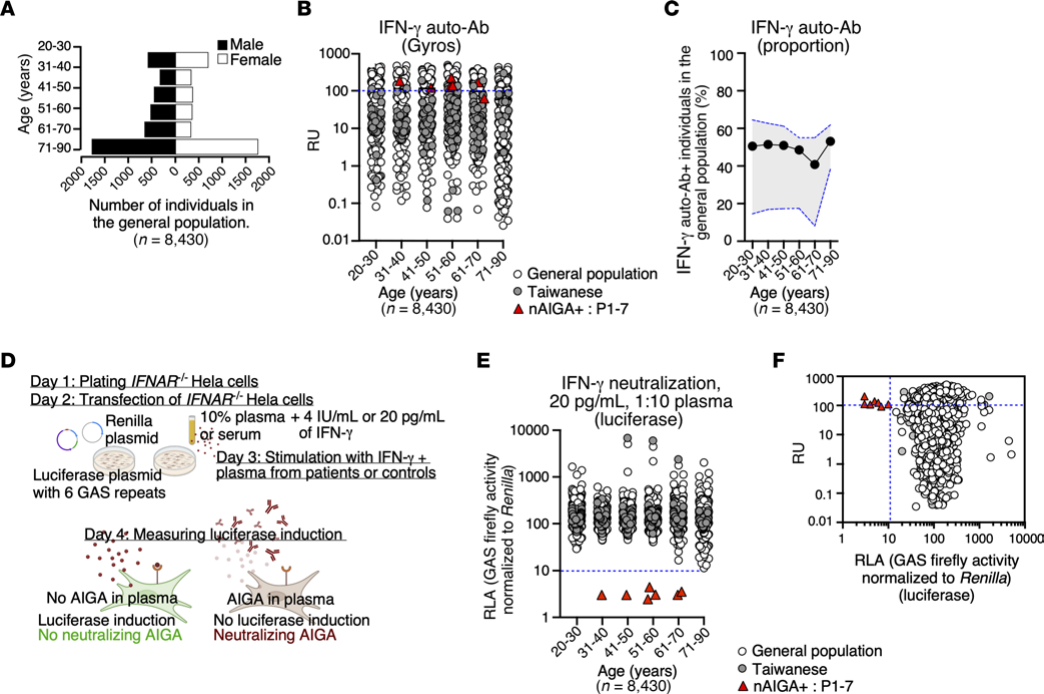
Nonneutralizing IFN-γ auto-Abs are detected in the general population. (**A**) Age and sex distribution of individuals from different cohorts are depicted in a bar plot. These cohorts include the French Blood Bank (EFS), 3C cohort, French CONSTANCES cohort, Cerba Healthcare cohort, and a cohort of Taiwanese healthy donors. (**B**) The detection of IFN-γ auto-Abs in the general population (white circles) compared with Taiwanese healthy donors (grey circles) and patients infected with EM and harboring nAIGAs, represented by red triangles, is shown in a dot plot. The detection thresholds are determined by auto-Abs against IFN-γ from patients with EM due to nAIGA (blue dotted line), measured in response units (RU). (**C**) Proportions of individuals positive for IFN-γ auto-Abs detection by Gyros are presented by decade, with standard deviation outlined in grey and a blue dotted line. (**D**) A schematic representation of the neutralization assay developed in *IFNAR*^–/–^ HeLa cells using a luciferase system is provided. The assay involves stimulation of transfected cells with IFN-γ and measurement of luciferase induction. (**E**) Results of the neutralization assay show relative luciferase activity (RLA) in the presence of plasma from the general population, Taiwanese individuals, and patients with nAIGA. A threshold of RLA < 15% is considered neutralizing (blue dotted line). (**F**) The correlation between detection by Gyros (RU) and neutralization is shown, with RLA after stimulation with IFN-γ in the presence of plasma. Individuals from the general population, Taiwanese individuals, and patients with nAIGA are represented. For large-scale screening, each sample was tested once.

We first searched for auto-Abs against IFN-γ in a subsampling of 87 individuals in the general population and compared them with 3 patients with EM infections due to potent nAIGAs (Supplemental Table 1; supplemental material available online with this article; https://doi.org/10.1172/JCI178263DS1) (29, 39). Samples of the general population were collected from the French Blood Bank (EFS), 3-city (3C) cohort, French CONSTANCES cohort, Cerba Healthcare cohort, and a cohort of Taiwanese healthy donors. We observed a positive signal (> 0.35 background corrected optical density [OD]) by ELISA primarily in IgG (51 of the 87 individuals) and IgM (43 of the 87 individuals) isotypes, whereas IgA and IgE were rarely (5 of the 87 individuals and 0 of the 87 individuals, respectively) detected (Supplemental Figure 1A). Since we detected anti-IFN-γ IgG auto-Abs in a subsampling of those in the general population and reported neutralizing IFN-γ auto-Abs are exclusively of the IgG isotype (19, 22) we next sought to determine the prevalence of IgG IFN-γ auto-Abs in a larger sample of those in the general population. To do this, we used Gyros technology, a high-throughput ELISA for the detection of anti-IFN-γ IgG. We tested a large cohort of 8,430 individuals aged 20–90 years from the general population, with an equal distribution between sexes (Figure 1A). Consistent with previous reports, of the 8,430 individuals tested for the detection of IFN-γ auto-Abs, we found an overall prevalence of approximately 49.2% of the general population, regardless of age (Figure 1, B and C) (38). To validate these results, we compared the detection of IFN-γ auto-Abs of 375 individuals as determined by Gyros with that determined by ELISA and found consistency between the 2 methods (91.7%) (Supplemental Figure 1B). Further, we demonstrate specificity of the detection of IFN-γ auto-Abs in the general population by determining whether the plasma of those that harbored these auto-Abs could also bind IFN-α2 or BSA. Those plasma containing auto-Abs against IFN-γ only bound IFN-γ and not IFN-α2 nor BSA, confirming the specificity of the detection by ELISA (Supplemental Figure 1C). Additionally, through IgG purification we observe that only the IgG fraction bound to IFN-γ, further validating the detection of IFN-γ auto-Abs in the general population (Supplemental Figure 1D). Although we detect IgG IFN-γ auto-Abs in a sizeable proportion of individuals in the general population, they are at a markedly lower titer compared with the 3 patients with nAIGAs who were tested (Supplemental Figure 1, E–G).

### Auto-Abs neutralizing IFN-γ are rare in the general population.

Next, we assessed the functionality of plasma of individuals of the general population to neutralize low, physiological concentrations of IFN-γ in vitro. To do this, we designed a high-throughput luciferase assay in which we transfected IFNAR1^–/–^ HeLa cells with (a) a plasmid containing 6 γ-activated sequence (GAS) element repeats and a firefly luciferase reporter and (b) a plasmid encoding the *Renilla* luciferase, which serves as a normalization control. We stimulated these cells with recombinant IFN-γ in the presence of 10% plasma (plasma 1:10) from patients known to harbor nAIGAs or individuals of the general population. We then measured firefly luciferase induction and normalized against *Renilla* luciferase activity (Figure 1D). At odds with the results obtained from the detection of IFN-γ auto-Abs by ELISA, we found that no individual of the general population harbored IFN-γ auto-Abs capable of neutralizing even a low, physiological concentration of IFN-γ (20 pg/mL; i.e., 4 IU/mL) (Figure 1E-F). Thus, while low levels of IFN-γ auto-Abs are common in individuals of the general population regardless of age, they are not capable of neutralization.

### Neutralizing and nonneutralizing IFN-γ Auto-Abs have different isotypes and epitopes.

**Figure 2:**
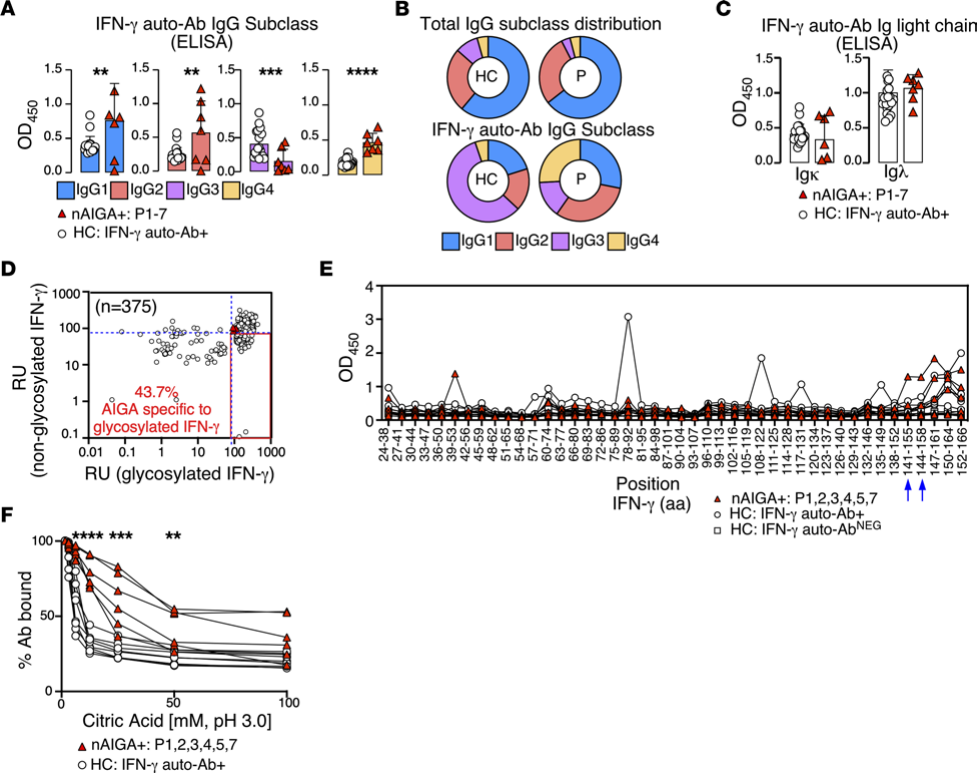
The common IFN-γ auto-Abs of the general population differ from patients with nAIGA. (**A**) Detection of anti-IFN-γ IgG subclasses from 20 individuals of the general population (white circles) and 7 patients with nAIGA (red triangles) is illustrated by ELISA, showcasing IgG1 (blue), IgG2 (red), IgG3 (purple), and IgG4 (yellow). (**B**) ELISA results display proportions of total IgG subclasses and anti-IFN-γ IgG from individuals of the general population and patients with nAIGA, highlighting the differences in subclass distribution. (**C**) Detection of anti-IFN-γ IgL from individuals of the general population and patients with nAIGA is shown by ELISA. (**D**) The correlation between detection of IFN-γ auto-Abs against glycosylated and nonglycosylated IFN-γ is depicted for individuals of the general population and patients with nAIGA. (**E**) Detection of linear peptides of IFN-γ from patients with nAIGA, individuals of the general population negative for detection against full-length IFN-γ auto-Abs, and individuals of the general population positive for detection against full-length IFN-γ auto-Abs is represented. Optical densities are plotted with respect to the amino acid position of IFN-γ. (**F**) Detection of high-affinity IFN-γ auto-Abs by ELISA with acid elution is shown, indicating citric acid concentration with respect to the percentage of bound IFN-γ auto-Ab remaining from patients with nAIGA and individuals of the general population positive for detection of IFN-γ auto-Abs. Data are representative of 2 independent experiments (**A**–**C**, **E**, and **F**), with each sample tested once for **D**. Statistical significance was calculated using an unpaired 2-tailed student’s *t* test, *P < 0.05, **P ≤ 0.01, ***P ≤ 0.001, ****P ≤ 0.0001.

To further profile IFN-γ auto-Abs in the general population, we determined the anti-IFN-γ IgG subclass and associated Ig light chain (IgL). Consistent with previous reports, we found a disproportionate level of anti-IFN-γ IgG4 in patients harboring nAIGAs relative to the normal ranges of total concentrations IgG4, whereas healthy individuals from the general population harbor nonneutralizing anti-IFN-γ IgG3 (Figure 2, A and B) (19). Further, IFN-γ auto-Abs of the patients and healthy individuals in the general population preferentially use Ig-λ, the infrequently used IgL (Figure 2C). Given that Abs can recognize carbohydrate or glycosylation sites on antigens (40) and that the prevalence of detectable IFN-γ auto-Abs in the general population is high, we hypothesized that IFN-γ auto-Abs in the general population specifically target glycosylated IFN-γ. To test this hypothesis, we assessed IFN-γ auto-Abs binding to glycosylated or nonglycosylated IFN-γ by Gyros. Consistent with our hypothesis, among the 375 healthy individuals of the general population tested, 164 individuals (43.7%) required glycosylated IFN-γ for IFN-γ auto-Ab recognition. In contrast, glycosylation is dispensable for the 3 patients carrying nAIGAs tested to recognize IFN-γ (Figure 2D).

### IFN-γ auto-Abs of the general population are of low affinity and are functionally distinct.

We next hypothesized that the IFN-γ auto-Abs of the general population recognized a different epitope than nAIGAs. Previously, it has been shown that nAIGAs target a major linear IFN-γ epitope, ‘SPAAKTGKRKR’, where the conserved ‘KRKR’ motif of the C-terminus of IFN-γ is required for bioactivity (41). To determine the specificity of the IFN-γ auto-Abs of the general population, we generated an overlapping peptide array derived from IFN-γ. Consistent with our hypothesis, we observed those of the general population that harbored auto-Abs to IFN-γ did not recognize the stereotyped ‘SPAAKTGKRKR’ IFN-γ motif (aa 144–154) (41), although 4 of the 7 individuals of the general population tested bound peptides of the C-terminus (Figure 2E). Further, among the 6 patients tested with nAIGA, only 1 recognized the major linear IFN-γ epitope, consistent with prior reports that some nAIGAs specifically bind to discontinuous, conformational epitopes (42). To corroborate these results we validated the specificities of the IFN-γ auto-Abs found in the general population and the nAIGAs found in the patients by PepperPrint, a custom epitope mapping peptide microarray. Consistent with our earlier results, we observed both patients and individuals of the general population bound peptides from the C-terminus of IFN-γ (Supplemental Figure 2A). Further, and concordant with our previous results, a single patient with nAIGAs recognized the major linear IFN-γ epitope ‘SPAAKTGKRKR’, while the other patient tested could recognize other peptides of the C-terminus containing the conserved ‘KRKR’ motif (Supplemental Figure 2B). Additionally, a single individual of the general population harbored auto-Abs that could significantly bind peptides ‘EDMNVKFFNSNKKKR’ (Supplemental Figure 2C). Next, we hypothesized that auto-Abs to IFN-γ in the general population would be of lower affinity relative to nAIGA. To test this hypothesis, we modified an ELISA to elute low affinity antibody with increasing doses of weak acid. Consistent with our hypothesis, we observed significantly less remaining IFN-γ auto-Abs bound upon acid elution from individuals of the general population relative to the nAIGA patients (Figure 2F). Therefore, the prevalent IFN-γ auto-Abs of the general population are functionally distinct with regards to affinity and IgG subclass.

### Low penetrance of nAIGAs in HLA-DRB1*15:02 and/or 16:02 carriers.

**Figure 3:**
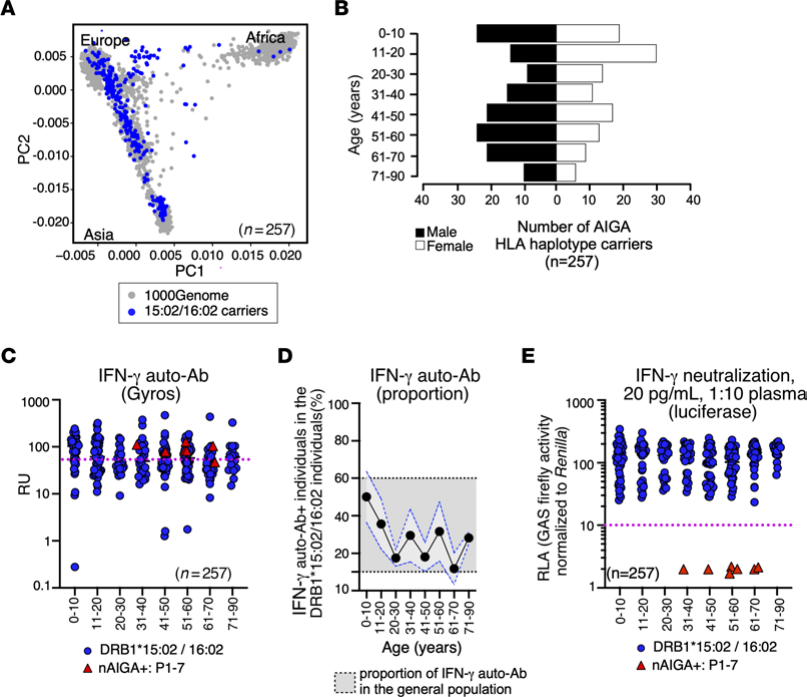
Low penetrance of nAIGAs in people who are carriers of HLA-DRB1*15:02 and/or 16:02. (**A**) PCA of 257 individuals carrying HLA-DRB1*15:02 and/or 16:02 alleles (blue). (**B**) Bar plot of the age and sex distribution of the individuals identified as carrying HLA-DRB1*15:02 and/or 16:02 alleles (*n* = 257). (**C**) Dot plot of the detection of IFN-γ auto-Abs in the HLA-DRB1*15:02 and/or 16:02 carriers (blue circles) and patients infected with EM and harboring nAIGA (red triangles) by Gyros. Positive threshold determined by detection of auto-Abs against IFN-γ from patients with EM due to nAIGA for each experiment (pink dotted line). Data are shown as RU. (**D**) Proportions by decade of those individuals positive for the detection of IFN-γ auto-Abs by Gyros. SD for detection of HLA-DRB1*15:02 and/or 16:02 carriers is shown in light grey with blue dotted line. Upper and lower threshold of the SD for the detection of IFN-γ auto-Abs in the general population is shown in dark grey with black dotted line. (**E**) Results for the neutralization of IFN-γ (20 pg/mL final concentration) in the presence of plasma 1:10 from HLA-DRB1*15:02 and/or 16:02 carriers and patients with nAIGA (red triangles). Relative luciferase activity is shown (GAS luciferase activity, with normalization against *Renilla* luciferase activity) after stimulation with IFN-γ (20 pg/mL final concentration) in the presence of 10% plasma. RLA, relative luciferase activity. For large-scale screening each sample was tested once.

Given that the HLA-DRB1*15:02 and/or 16:02 alleles are strongly associated with the development of nAIGAs (29, 30), we next determined the penetrance of nonneutralizing IFN-γ auto-Abs and nAIGAs in HLA-DRB1*15:02 and/or 16:02 carriers. We first determined the HLA-DRB1 haplotypes from a cohort of 23,769 whole exome sequences (WES). We identified 1,644 individuals carrying HLA-DRB1*15:02 and/or 16:02 alleles. We observed that the HLA-DRB1*15:02 and/or 16:02 carriers are primarily stratified among individuals of European or Asian descent (Figure 3A). Blood samples were collected from 257 HLA-DRB1*15:02 and/or 16:02 carriers, who were aged 0–90 years with an equal distribution between sexes (Figure 3B). We found that 71 HLA-DRB1*15:02 and/or 16:02 carriers had detectable IFN-γ auto-Abs by Gyros (approximately 27.6%) regardless of age, which was within the range of the prevalence of IFN-γ auto-Abs detected in the general population (Figure 3, C and D). We next assessed the functionality plasma of the HLA-DRB1*15:02 and/or 16:02 carriers to neutralize low, physiological levels of IFN-γ and found that none of the HLA-DRB1*15:02 and/or 16:02 carriers could neutralize 20 pg/mL of IFN-γ (Figure 3E). Therefore, the penetrance of nAIGAs was very low in HLA-DRB1*15:02 and/or 16:02 carriers (< 0.012, CI=95%; < 0.025, CI=95% for those over 40 yrs), suggesting that rare germline or somatic variants drive the development of nAIGA. Moreover, HLA-DRB1*15:02 and/or 16:02 do not increase the likelihood of nonneutralizing auto-Abs against IFN-γ, further dissociating the 2 types of auto-Abs. Thus, HLA-DRB1*15:02 and/or 16:02 may be strongly associated with, yet are insufficient for, the development of nAIGA.

### nAIGAs are rare in patients with autoimmune conditions.

**Figure 4:**
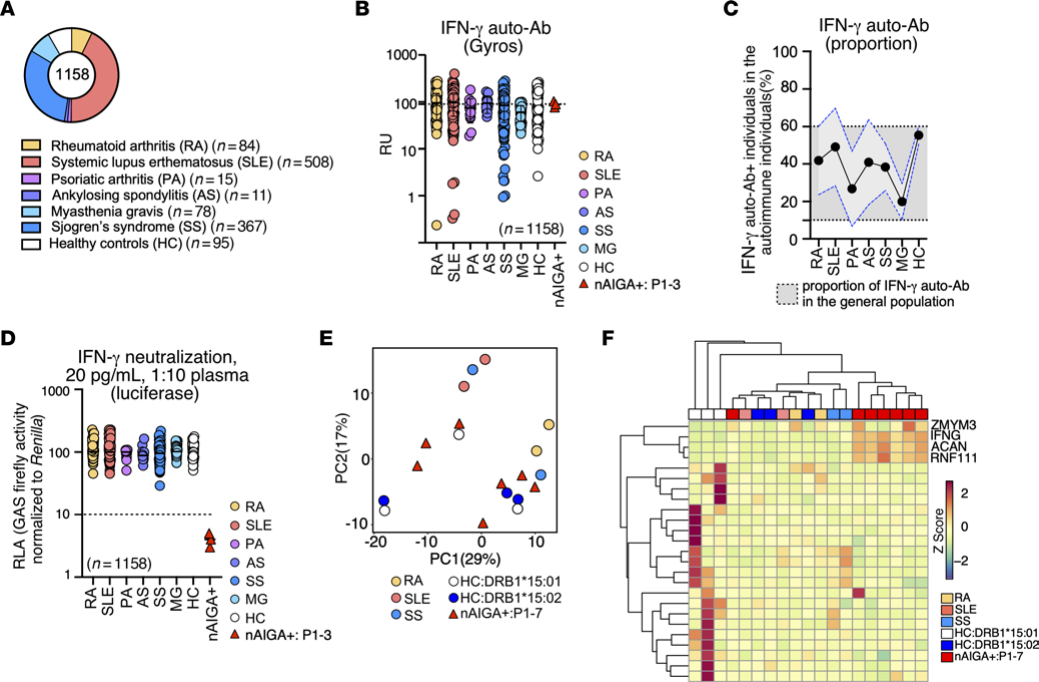
nAIGAs are rare and are not detected in those with autoimmune conditions. (**A**) Distribution of samples collected from Taiwanese individuals with various autoimmune diseases, including rheumatoid arthritis (RA, *n* = 84) (yellow), systemic lupus erythematosus (SLE, *n* = 508) (red), psoriatic arthritis (PS, *n* = 15) (pink), ankylosing spondylitis (AS,*n* = 11) (purple), and Sjogren’s syndrome (SS, *n* = 367) (blue), in addition to healthy controls (white, *n* = 95). Those with myasthenia gravis (MG, *n* = 78) (light blue) are predominantly European. (**B**) Detection of IFN-γ auto-Abs by Gyros in individuals with different autoimmune diseases and healthy controls is shown, with a positive threshold determined by nAIGA detection in patients with EM. (**C**) Proportions of individuals positive for IFN-γ auto-Ab detection by Gyros are depicted by autoimmune disease, with SD shown for autoimmune patients. (**D**) Results of IFN-γ neutralization assay in the presence of plasma from autoimmune patients, healthy controls, and patients with nAIGA are displayed, showing relative luciferase activity after stimulation with IFN-γ. (**E**) Principal component analysis (PCA) of the autoimmune humoral repertoire of patients with nAIGA, RA, SLE, SS, HLA-DRB1*15:02 and/or 16:02 carriers, and HLA-DRB1*15:01 carriers is shown. (**F**) Heatmap and hierarchical clustering of top autoantibody specificities from patients with nAIGA, RA, SLE, SS, HLA-DRB1*15:02 and/or 16:02 carriers, and HLA-DRB1*15:01 carriers are presented. Each sample was tested once for large-scale screening. This data underscores the rarity of nAIGA and their distinct absence in autoimmune conditions.

A number of patients with nAIGAs display comorbid conditions including endocrine disorders, cancers, and autoimmune diseases (24, 43, 44). Additionally, auto-Abs against IFN-α/ω have been reported in patients with systemic lupus erythematous (SLE) (45, 46), Sjogren’s syndrome (SS) (46), thymoma (47), or myasthenia gravis (MG) (47–49). Given that neutralizing auto-Abs to IFN-γ appear to be exceedingly rare and that HLA-DRB1*15:02 and/or 16:02 alleles are strongly associated but not sufficient for their development, we hypothesized that patients with autoimmune conditions often observed among those harboring nAIGAs may also carry such autoantibodies, even in the absence of EM disease. Samples were collected from Taiwanese patients with rheumatoid arthritis (RA) (*n* = 84), SLE (*n* = 508), psoriatic arthritis (PS) (*n* = 15), ankylosing spondylitis (AS) (*n* = 11), and SS (*n* = 367). Additionally, we included a cohort of MG patients predominantly of European descent (*n* = 78) (Figure 4A). We found that 38 of the 84 RA (45.2%), 272 of the 508 SLE (53.5%), 6 of the 15 PS (40%), 5 of the 11 AS (45.4%), 156 of the 367 SS (43.3%), and 22 of the 97 MG (22.6%) patients had detectable IFN-γ auto-Abs by Gyros, which was within the range of the prevalence of IFN-γ auto-Abs detected in the general population (Figure 4, B and C). We next found that none of these patient’s plasma neutralized IFN-γ (Figure 4D). Thus, these data suggest that nAIGAs are rare in patients with autoimmune conditions that can be associated with them.

### nAIGAs are not associated with other auto-Abs.

Since our data suggest that nAIGAs are rare even in healthy individuals and patients with autoimmunity, we hypothesized that the patients with nAIGAs may have a distinctive autoimmune Ab repertoire. First, we tested whether there were aberrations in serum levels of total immunoglobulin (Ig) in 7 patients with nAIGAs capable of neutralizing even a high concentration of IFN-γ (20 ng/mL), when compared with age-, ethnic-, and HLA-DRB1-matched controls (Supplemental Figure 3A and Supplemental Table 1) (28, 38). We found that there were no obvious perturbations of total Ig and the patients were within the normal range for total Ig (Supplemental Figure 3B). Next, we analyzed the serum from the 7 patients with nAIGAs by HuProt, a comprehensive human proteome microarray containing over 21,000 human protein isoforms. Globally, the autoimmune humoral repertoire of the 7 patients did not cluster relative to 3 ethnic and aged-matched healthy controls carrying HLA-DRB1*15:02, 3 ethnic and aged-matched healthy controls carrying HLA-DRB1*15:01, nor to those with autoimmune conditions (RA, SLE, and SS) (Figure 4E). In general, there were no more autoreactivities in patients than in controls, nor in patients with autoimmunity. Nevertheless, we found a few auto-Abs in the patients harboring nAIGAs but not in controls, nor did we find them in the few autoimmune patients tested (Figure 4F). These auto-Abs were against ACAN, a known biomarker in RA (50, 51) and ZMYM3 and RNF111, implicated in chronic lymphocytic leukemia (52, 53) and cancers (54), respectively. Consistent with these results, we found relatively few auto-Abs in the 7 patients with nAIGAs tested by a complementary approach, Phage Immunoprecipitation–Seq (PhIP-Seq) (Supplemental Figure 3C). Therefore, nAIGAs are apparently distinctive, rare, and isolated.

### nAIGAs are rare in patients with mycobacterial disease.

**Figure 5:**
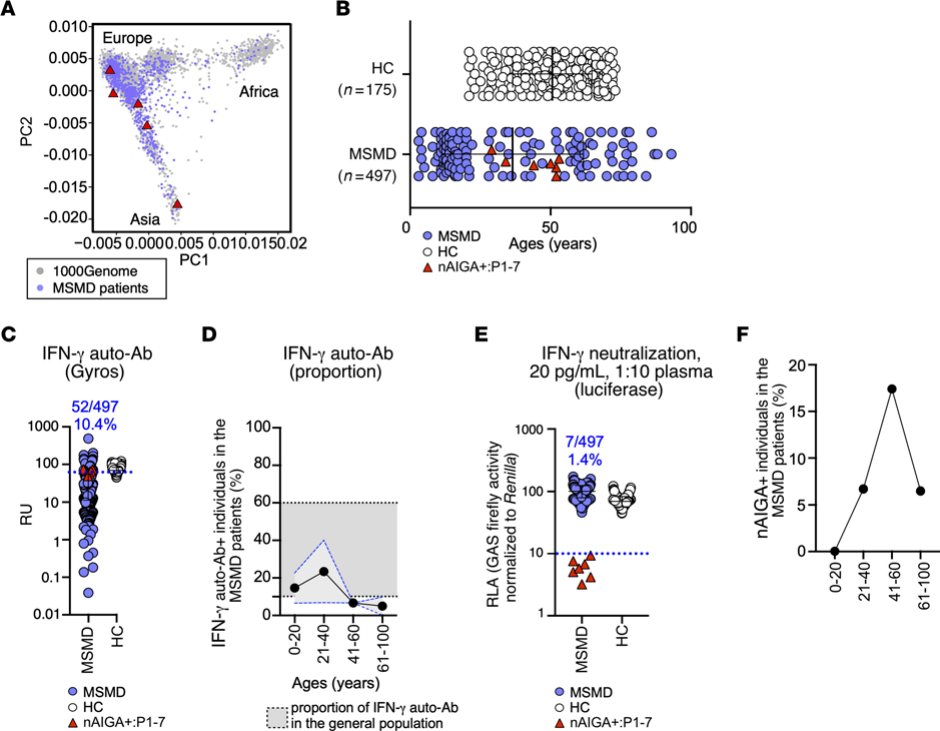
nAIGAs are not detected in those with unexplained, severe disease due to mycobacteria. (**A**) PCA of patients with unexplained mycobacteria infections (purple circles). (**B**) Dot plot of the age distribution of the patients with unexplained mycobacteria infections (*n* = 497) and healthy controls (*n* = 175). (**C**) Dot plot of the detection of IFN-γ auto-Abs in patients with unexplained mycobacteria infections (*n* = 490) (purple circles), and healthy controls (HC, *n* = 175) (white circles) and patients infected with EM and harboring 7 nAIGA (red triangles) by Gyros. Positive threshold determined by detection of auto-Abs against IFN-γ for each experiment (black dotted line). Data are shown in RU. (**D**) Proportions by age of patients positive for the detection of IFN-γ auto-Abs by Gyros. SD for detection for patients with MSMD is shown in light grey with blue dotted line. Upper and lower threshold of the SD for the detection of IFN-γ auto-Abs in the general population is shown in dark grey with black dotted line. (**E**) The neutralization of IFN-γ (20 pg/mL) with plasma 1:10 from mycobacteria-infected patients (purple circles) and nAIGA patients (red triangles) is depicted. Relative luciferase activity after IFN-γ stimulation is shown (GAS luciferase activity normalized against Renilla luciferase activity). (**F**) Proportions by age of patients positive for the detection of nAIGA as shown in **E**. For large-scale screening, each sample was tested once.

Potent nAIGAs predispose individuals to infection with EM. Despite the relevance of a diagnosis of nAIGAs in patients with EM, if only to exclude a search of an MSMD genetic etiology, the prevalence of nAIGAs is unknown in patients with unexplained, severe disease due to mycobacteria. Therefore, we next assessed in a sample of 497 patients with mycobacterial disease without any known MSMD genetic etiology. These patients typically segregated among European populations (Figure 5A) and were aged 0–93 years (Figure 5B). We found that 52 of the 497 patients tested had detectable IFN-γ auto-Abs by Gyros (10.4%), which was somewhat lower than the prevalence of IFN-γ auto-Abs detected in the general population (Figure 5, C and D). Endogenous IFN-γ may perhaps be bound to the circulating IFN-γ auto-Abs, thereby blocking detection by Gyros. Alternatively, the circulating humoral repertoire could be skewed to mycobacterium-specific antibodies. We next determined whether the plasma of these patients could neutralize a low physiological concentration of IFN-γ and we found that 7 of these 497 patients (1.4%) harbored nAIGAs (Figure 5E). Consistent with previous reports, those aged between 41–60 years had the highest proportions of nAIGA^+^ patients (17.4%) (Figure 5F). Further, and consistent with studies showing a strong association of the HLA-DRB1*15:02 or 16:02 with nAIGAs (29, 30), 5 of the 7 (71.4%) patients carry those class II alleles. One of the remaining 2 patients carried HLA-DRB1*15:01 and HLA-DRB1*16:01 alleles, which are closely related to HLA-DRB1*15:02 and 16:02, respectively. The remaining nAIGA^+^ patients carried HLA-DRB1*13:01. Despite those few nAIGA+ patients, nAIGAs were unfound in 41 patients who have HLA-DRB1*15:02 or 16:02 (< 0.071, CI = 95%) nor in 45 patients with IFN-γ auto-Abs, nor in 5 patients with both, which exemplifies the rarity of nAIGAs. Thus, nAIGAs are rare (1.4%) even among patients with unexplained, severe disease due to mycobacteria, including HLA-DRB1*15:02 or 16:02 patients, and are therefore unlikely to be driven by the infection.

## Discussion

We confirm that auto-Abs to IFN-γ are relatively common in the general population and show that potent nAIGAs are rare. nAIGAs are not found in the general population and even in HLA-DRB1*15:02 and/or 16:02 individuals. Their penetrance in HLA-DRB1*15:02 and/or 16:02 is therefore very low. While HLA-DRB1*15:02 and/or 16:02 are strongly associated, they are insufficient for the development of nAIGA. This suggests that there may be rare germline or somatic variants triggering the development of nAIGA. The rarity of familial cases of infectious diseases due to nAIGAs suggests incomplete penetrance of inherited mutations, or the responsibility of de novo or somatic variants. Given that the development of nAIGAs necessitates HLA-DRB1*15:02 and/or 16:02 alleles and that auto-Abs to other cytokines can be genetically driven by mutations underlying defects in T cell development (55–57) and function (58, 59), it is conceivable that inborn or somatic errors of T cell defects underlie nAIGA. Alternatively, rare variants intrinsic to peripheral, tolerogenic antigen presenting cells (60) or the antibody-producing B cell precursors themselves could underlie the development of nAIGA. Screening of patients with inborn errors of immunity who carry either at-risk HLA-DRB1 allele is warranted.

Peptide scanning revealed that the major linear B cell epitope (aa 144–154) in the carboxy-terminal of IFN-γ is targeted by nAIGAs, and is required for the optimal neutralization of IFN-γ (41, 61). Nevertheless, it has been shown that other nAIGAs can recognize conformational, discontinuous epitopes targeting helical C and E regions near His 19/20 (42). Our data show that the auto-Abs to IFN-γ detected in the general population do not recognize the previously described ‘SPAAKTGKRKR’ motif. However, these auto-Abs to IFN-γ in the general population can recognize peptides of the C-terminus of IFN-γ, but only with low affinity, low titer, and eliciting a different effector function by their preferred IgG subclass. Although the function of these common auto-Abs to IFN-γ remain unknown, they may abrogate the activity of IFN-γ, albeit only marginally, given their low titer and affinity. Alternatively, the common auto-Abs to IFN-γ may act synergistically with or extend the half-life of IFN-γ. Given the rarity and specificity of nAIGAs, their emergence is unlikely to be preceded by their nonneutralizing counterparts, as the B cells responsible for the former are improbable progenitors of the latter. Conversely, given that both nonneutralizing and neutralizing auto-Abs can target peptides of the C-terminus of IFN-γ, it is conceivable that nAIGAs emanate exclusively from B cells experiencing substantial expansion and affinity maturation. Further studies are necessary to determine the consequences of nonneutralizing auto-Abs and the origins of nAIGAs.

MSMD is rare, with a prevalence estimated to be between 10^–5^–10^–4^ in the general population (16). Similarly, we have shown that nAIGAs are perhaps correspondingly as rare, therefore further implicating their causal role in infections with EM. Furthermore, the most frequent clinical manifestation of patients harboring nAIGAs are severe infections with EM. All patients with nAIGAs reported in this current study had infections with EM, therefore it is probable that nAIGAs are pathogenic with high and perhaps complete penetrance. However, some patients have been observed to have been infected with isolates of *B*. *cocovenenans* (21), *T*. *marneffei* (25), and even *M*. *tuberculosis* (18) in the years prior to developing an infection with EM. Further, it has been reported that 1 ART-responsive, HIV-infected patient harbored nAIGAs 18 months before disseminated *M*. *avium* complex infection, providing evidence of preexisting nAIGAs (62). Potent nAIGAs might develop preferentially after continued exposure to IFN-γ. Alternatively and more likely, the auto-Abs might have contributed to the development of earlier mycobacterial or opportunistic infections. None of the individuals with detectable, nonneutralizing auto-Abs against IFN-γ had mycobacterial disease. Overall, our findings confirm that nAIGAs underlie EM disease, establish a very low penetrance in HLA-DRB1*15:02 and/or 16:02 individuals, and emphasize the rarity and isolation of nAIGAs.

## Methods

### Sex as a biological variable.

We enrolled large-scale population samples with age and sex distribution reported for each respective cohort. Our study enrolled both sexes equally distributed across age by decade. No sex bias has been reported for the development nAIGAs, thus it is an unlikely variable, however this variable was assessed and reported.

### Screening of auto-Abs to IFN-γ by Gyros.

Recombinant human (rh) IFN-γ (R&D Systems, 285-IF-100/CF or 10067-IF-100) was first biotinylated with EZ-Link Sulfo-NHS-LC-Biotin (Thermo Fisher Scientific, A39257), according to the manufacturer’s instructions, with a biotin-to-protein molar ratio of 1:12. The detection reagent contained a secondary antibody (Alexa Fluor 647 goat anti-human IgG [Thermo Fisher Scientific, A21445]) diluted in Rexxip F (Gyros Protein Technologies, P0004825; 1:500 dilution of the 2 mg/mL stock to yield a final concentration of 4 μg/mL). Buffer phosphate-buffered saline, 0.01% Tween 20 (PBS-T) and Gyros Wash buffer (Gyros Protein Technologies, P0020087) were prepared according to the manufacturer’s instructions. Plasma or serum samples were then diluted 1:100 in 0.01% PBS-T and tested with the Bioaffy 1000 CD (Gyros Protein Technologies, P0004253) and the Gyrolab xPand (Gyros Protein Technologies, P0020520). Cleaning cycles were performed in 20% ethanol. Threshold for positivity was determined by the average of the positive controls run on the day of the experiment in each run.

### Screening of auto-Abs to IFN-γ by ELISA and peptide epitope mapping.

96-well ELISA plates (MaxiSorp, Thermo Fisher Scientific) were coated by incubation overnight at 4°C with IFN-γ (5 μg/mL; Imukin, R&D Systems, 285-IF-100/CF, 10067-IF-100). Plates were then washed with PBS, 0.01% Tween 20, blocked by incubation with 10% BSA in the same buffer overnight. Plates were then washed and incubated with 1:25 dilutions of plasma from the patients or controls for at least 12 hours at 4°C. Plates were thoroughly washed. HRP–conjugated Fc-specific IgG fractions from polyclonal goat antiserum against human IgG (1:10,000; Jackson ImmunoResearch, 109-035-008), IgM(1:10,000; Jackson ImmunoResearch, 109-035-043), or IgA (1:10,000; Jackson ImmunoResearch, 109-035-011) or IgG1 (1:5,000; SourthernBiotech, 9054-05), IgG2 (1:5,000; SouthernBiotech, 9060-05), IgG3 (1:5,000; SouthernBiotech, 9210-05), or IgG4 (1:5,000, SouthernBiotech, 9200-05). For the assessment of light chain usage, HRP-conjugated polyclonal goat antiserum against human Ig-λ (1:5,000; Sigma-Aldrich, AP506P) and Ig-κ (1:5,000; Sigma-Aldrich, AP502P) were added after the incubation with the plasma. Plates were then incubated for 2 hours at room temperature and washed. Substrate was added, and the OD was measured. To determine the affinity of IFN-γ auto-Abs, standard ELISA included weak acid elution (0.5M citric Acid, pH = 3.0) after incubation with plasma for 15 minutes. All samples to be assessed for affinity were normalized for the amount of specific antibody present in the sample, therefore all samples were previously titrated and diluted appropriately. Detection of linear epitopes was determined by the generation of a peptide library (Vivitide, custom library), 15-mer peptides with an 11-amino acid overlap derived from IFN-γ. Peptides were plated at 5 μg/mL on Nunc immobilizer amino acid plates (Thermo Scientific; 436006) overnight at 4°C. Standard ELISA procedure follows as a above. For validation of the epitope mapping, PepperPrint is a commercially available peptide microarray (63).

### Screening of auto-Abs to IFN-γ by luciferase assay.

The blocking activity of IFN-γ auto-Abs was determined with a reporter luciferase activity. Briefly, *IFNAR1*^–/–^ HeLa cells were transfected with a plasmid containing the firefly luciferase gene under the control of 6 tandem repeats of human GAS promoter and a plasmid constitutively expressing *Renilla* luciferase for normalization (Qiagen, Cignal reporter assay kits, CCS-009L). Cells were transfected in the presence of the Lipofectamine LTX and Plus transfection reagent (Invitrogen, 15338-100) for 24 hours. Cells in DMEM (Thermo Fisher Scientific) supplemented with 5% FBS and 10% healthy control or patient serum/plasma (after inactivation at 56°C for 20 minutes) were stimulated with 20 pg/mL IFN-γ (Imukin, 9661191534306) for 16 hours at 37°C. Finally, cells were lysed for 10 minutes at room temperature, and luciferase levels were measured with the Dual-Glo Luciferase Assay System (Promega, E2940) according to the manufacturer’s protocol. Luminescence intensity was measured with a SpectraMax Id3 (Molecular Devices). Firefly luciferase activity values were normalized against Renilla luciferase activity values. These values were then normalized against the fold change induction of pooled healthy serum (Sigma-Aldrich, H4522-20ML) in the presence of 20 pg of IFN-γ relative to nonstimulated, pooled healthy serum condition. Samples were considered neutralizing if luciferase induction, normalized against *Renilla* luciferase activity, was below 10% of the mean values for controls tested the same day.

### PhIP-Seq.

PhIP-Seq was performed following our previously published vacuum-based PhIP-Seq protocol (64). (https://www.protocols.io/view/scaled-high-throughput-vacuum-phip-protocol-ewov1459kvr2/v1).

### PhIP-Seq analysis.

All analysis (except when specifically stated otherwise) was performed at the gene level. Reads for all peptides mapping to the same gene were summed, and 0.5 reads were added to each gene. Within each individual sample, reads were normalized by converting to the percentage of total reads. To normalize each sample against background nonspecific binding, a FC over mock-IP was calculated by dividing the sample read percentage for each gene by the mean read percentage of the same gene for the AG bead–only controls. To identify those auto-Abs specifically enriched in patients with MSMD, Z-scores were calculated for each auto-Ab in each patient relative to 66 healthy controls (60 HLA-DRB1 unknown, 3 HLA-DRB1*15:01, 3 HLA-DRB1*15:02). Consistent with our previous studies (64) antibodies with a Z-score greater than 10 over controls were determined as positive.

### Protein microarray.

Protein microarrays from CDI laboratories (HuProt) underwent a 90-minute incubation in 5 mL blocking buffer, composed of 2% BSA and 0.05% Tween-20 in PBS. Following that, arrays were left overnight in 5 mL blocking buffer per array, with serum from a donor or patient at a 1:2,000 dilution. Each array underwent a series of 5 5-minute washes with 5 mL PBS-T (PBS + 0.05% Tween-20). Alexa Fluor 647 goat anti-human IgG (Thermo Fisher Scientific, A-21445, RRID:AB 2535862) and Dylight 550 goat anti-GST (Columbia Biosciences, D9-1310) were diluted in blocking buffer (1:2,000 and 1:10,000, respectively), and each array was incubated for 90 minutes in 5 mL of the resulting mixture. Subsequently, 5 washes were performed as described earlier. Incubations and washes occurred on an orbital shaker, with aluminum foil used to block light after adding fluorescent antibodies. Finally, each array underwent 3 immersions in deionized water and centrifugation for approximately 30 seconds to dry. Later the same day, arrays were scanned using an Innoscan 1100AL Fluorescence scanner (Innopsys) with Mapix v.9.1.0. Normalization compensated for signal intensity variation between experiments. Data from additional healthy blood donors of separate protein array experiments using the same protocol were incorporated. Signal intensities were extracted from scanned images using GenePix Pro v.5.1.0.19 and GenePix Pro 7, involving the subtraction of the local background.

### Statistics.

Statistical details of all experiments including tests used, n, and number of experimental repeats are provided in figure legends. Briefly, 2-tailed unpaired student’s *t* tests were used to determine statistical significance where appropriate. Prism graphpad (v9.3.0) was used for statistical analyses and graphing. R studio (v3.6.1) was used for the visualization of PCA and Z-scores for HuProt and PhIP-Seq.

### Study approval.

We enrolled 8,430 people who were healthy controls, 257 people who were HLA-DRB1*15:02 or 16:02 carriers, and 497 patients with MSMD. The healthy controls were recruited in collaboration with CONSTANCES cohort, 3C-Dijon Study, Cerba Health-Care, Etablissement du Sang study group, and with Cheng Lung Ku at Chang Gung University. The 257 HLA-DRB1*15:02 or 16:02 carriers and 497 patients with MSMD were enrolled through St. Giles Laboratory of Human Genetics of Infectious Diseases and HLA type and ethnicity are inferred through WES. All individuals were recruited according to protocols approved by local IRBs. Written informed consent was obtained in the country of residence of each patient. Experiments were conducted in France and the United States in accordance with local regulations and with the approval of the IRB of the Institut National de la Santé et de la Recherche Médicale and the Rockefeller University, respectively. Approval was obtained from the French Ethics Committee (Comité de Protection des Personnes), the French National Agency for Medicine and Health Product Safety, the Institut National de la Santé et de la Recherche Médicale in Paris, France (protocol no. C10-07), and the Rockefeller University IRB in New York, New York, USA (protocol no. JCA-0699).

### Data availability.

All data are available within the Supporting data values file. Plasma, cells, and genomic DNA are available from JLC under a MTA with The Rockefeller University. Materials and reagents used are almost exclusively commercially available and nonproprietary. Requests for materials derived from human samples may be made available, subject to any underlying restrictions on such samples. JLC can make MTAs available through The Rockefeller University.

## Author contributions

JNP, RY, TLV, AC, A Bodansky, A Behere, MSA, NL, LA, J Bustamante , SBD, CLK, and JLC, performed or provided supervision of experiments, generated and analyzed data, and contributed to the manuscript by providing figures and tables. JNP, AC, A Bodansky, A Behere, YS, and CC, performed computational analysis of data. JYD, WTL, LB, CS, MM, TP, LP, F Martin, F Messina, JS, PB, PT, SMS, RG, LK, MB, EL, LT, CG, and colloborators of CONSTANCES cohort, 3C-Dijon Study, Etablissement du Sang study group evaluated and recruited patients and/or control cohorts of patients, or healthy controls. JNP and JLC wrote the manuscript. JLC, SBD, and J Bustamante supervised the project. MO, AY, DL, J Bohlen, JR, AP, AG, and EJ contributed to invaluable discussion. All authors edited the manuscript. RY and TLV share second authorship and contributed equally to this work. AG, JR, and PB contributed equally to this work as co–third authors. A Behere, AC, and A Bodansky share fourth authorship and contributed equally to this work. PT, LA, SBD, J Bustamante, and CLK equally contributed to this work.

## Supplementary Material

Supplemental data

ICMJE disclosure forms

Supplemental data set 1

Supporting data values

## Version 1 03/12/2024

In-Press Preview

## Version 2 04/15/2024

Electronic publication

## References

[1] Casanova JL (1995). Immunological conditions of children with BCG disseminated infection. Lancet.

[2] Casanova JL, Abel L (2002). Genetic dissection of immunity to mycobacteria: the human model. Annu Rev Immunol.

[3] Poyhonen L (2019). Life-threatening infections due to live-attenuated vaccines: early manifestations of inborn errors of immunity. J Clin Immunol.

[4] Newport M, Levin M (1994). Familial disseminated atypical mycobacterial disease. Immunol Lett.

[5] Casanova JL, Abel L (2021). Lethal infectious diseases as inborn errors of immunity: toward a synthesis of the germ and genetic theories. Annu Rev Pathol.

[6] Casanova JL, Abel L (2021). Mechanisms of viral inflammation and disease in humans. Science.

[7] Bustamante J (2020). Mendelian susceptibility to mycobacterial disease: recent discoveries. Hum Genet.

[8] Bohlen J (2023). Human MCTS1-dependent translation of JAK2 is essential for IFN-γ immunity to mycobacteria. Cell.

[9] Rosain J (2023). Human IRF1 governs macrophagic IFN-γ immunity to mycobacteria. Cell.

[10] Martin-Fernandez M (2022). A partial form of inherited human USP18 deficiency underlies infection and inflammation. J Exp Med.

[11] Yang R (2020). Human T-bet governs innate and innate-like adaptive IFN-γ immunity against mycobacteria. Cell.

[12] Neehus AL (2024). Human inherited CCR2 deficiency underlies progressive polycystic lung disease. Cell.

[13] Le Voyer T (2021). Inherited deficiency of stress granule ZNFX1 in patients with monocytosis and mycobacterial disease. Proc Natl Acad Sci U S A.

[14] Kerner G (2020). Inherited human IFN-γ deficiency underlies mycobacterial disease. J Clin Invest.

[15] Dupuis S (2000). Human interferon-gamma-mediated immunity is a genetically controlled continuous trait that determines the outcome of mycobacterial invasion. Immunol Rev.

[16] Casanova JL, Abel L (2022). From rare disorders of immunity to common determinants of infection: Following the mechanistic thread. Cell.

[17] Kampmann B (2005). Acquired predisposition to mycobacterial disease due to autoantibodies to IFN-gamma. J Clin Invest.

[18] Doffinger R (2004). Autoantibodies to interferon-gamma in a patient with selective susceptibility to mycobacterial infection and organ-specific autoimmunity. Clin Infect Dis.

[19] Browne SK (2012). Adult-onset immunodeficiency in Thailand and Taiwan. N Engl J Med.

[20] Zhang B (2023). Characteristics and outcomes of anti-interferon gamma antibody-associated adult onset immunodeficiency. J Clin Immunol.

[21] Hoflich C (2004). Naturally occurring anti-IFN-gamma autoantibody and severe infections with Mycobacterium cheloneae and Burkholderia cocovenenans. Blood.

[22] Puel A (2022). Human autoantibodies underlying infectious diseases. J Exp Med.

[23] Patel SY (2005). Anti-IFN-gamma autoantibodies in disseminated nontuberculous mycobacterial infections. J Immunol.

[24] Shih HP (2021). Anti-interferon-γ autoantibody-associated immunodeficiency. Curr Opin Immunol.

[25] Guo J (2020). Anti-IFN-γ autoantibodies underlie disseminated Talaromyces marneffei infections. J Exp Med.

[26] Chen ZM (2022). Anti-interferon-γ autoantibodies impair T-lymphocyte responses in patients with *Talaromyces marneffei* infections. Infect Drug Resist.

[27] Hong GH (2020). Natural history and evolution of anti-interferon-γ autoantibody-associated immunodeficiency syndrome in Thailand and the United States. Clin Infect Dis.

[28] Dendrou CA (2018). HLA variation and disease. Nat Rev Immunol.

[29] Ku CL (2016). Anti-IFN-γ autoantibodies are strongly associated with HLA-DR*15:02/16:02 and HLA-DQ*05:01/05:02 across Southeast Asia. J Allergy Clin Immunol.

[30] Chi CY (2013). Anti-IFN-γ autoantibodies in adults with disseminated nontuberculous mycobacterial infections are associated with HLA-DRB1*16:02 and HLA-DQB1*05:02 and the reactivation of latent varicella-zoster virus infection. Blood.

[31] Bastard P (2021). Autoantibodies neutralizing type I IFNs are present in ~4% of uninfected individuals over 70 years old and account for ~20% of COVID-19 deaths. Sci Immunol.

[32] Bastard P (2020). Autoantibodies against type I IFNs in patients with life-threatening COVID-19. Science.

[33] Alotaibi F (2023). Type I interferon autoantibodies in hospitalized patients with Middle East respiratory syndrome and association with outcomes and treatment effect of interferon beta-1b in MIRACLE clinical trial. Influenza Other Respir Viruses.

[34] Zhang Q (2022). Autoantibodies against type I IFNs in patients with critical influenza pneumonia. J Exp Med.

[35] Gervais A (2023). Autoantibodies neutralizing type I IFNs underlie West Nile virus encephalitis in approximately 40% of patients. J Exp Med.

[36] Bastard P (2021). Auto-antibodies to type I IFNs can underlie adverse reactions to yellow fever live attenuated vaccine. J Exp Med.

[37] Ratnatunga CN (2020). The rise of non-tuberculosis mycobacterial lung disease. Front Immunol.

[38] Caruso A (1990). Natural antibodies to IFN-gamma in man and their increase during viral infection. J Immunol.

[39] Valour F (2016). Interferon-γ autoantibodies as predisposing factor for nontuberculous mycobacterial infection. Emerg Infect Dis.

[40] Kappler K, Hennet T (2020). Emergence and significance of carbohydrate-specific antibodies. Genes Immun.

[41] Lin CH (2016). Identification of a major epitope by anti-interferon-γ autoantibodies in patients with mycobacterial disease. Nat Med.

[42] Shih HP (2022). Pathogenic autoantibodies to IFN-γ act through the impedance of receptor assembly and Fc-mediated response. J Exp Med.

[43] Chi CY (2016). Clinical manifestations, course, and outcome of patients with neutralizing anti-interferon-γ autoantibodies and disseminated nontuberculous mycobacterial infections. Medicine (Baltimore).

[44] Angkasekwinai N (2019). Clinical outcome and laboratory markers for predicting disease activity in patients with disseminated opportunistic infections associated with anti-interferon-γ autoantibodies. PLoS One.

[45] Panem S (1982). Antibodies to alpha-interferon in a patient with systemic lupus erythematosus. J Immunol.

[46] Gupta S (2016). Distinct functions of autoantibodies against interferon in systemic lupus erythematosus: a comprehensive analysis of anticytokine autoantibodies in common rheumatic diseases. Arthritis Rheumatol.

[47] Shiono H (2003). Spontaneous production of anti-IFN-alpha and anti-IL-12 autoantibodies by thymoma cells from myasthenia gravis patients suggests autoimmunization in the tumor. Int Immunol.

[48] Bello-Rivero I (2004). Characterization of the immunoreactivity of anti-interferon alpha antibodies in myasthenia gravis patients. Epitope mapping. J Autoimmun.

[49] Meager A (2003). Anti-cytokine autoantibodies in autoimmunity: preponderance of neutralizing autoantibodies against interferon-alpha, interferon-omega and interleukin-12 in patients with thymoma and/or myasthenia gravis. Clin Exp Immunol.

[50] Karopoulos C (1996). Presence of antibodies to native G1 domain of aggrecan core protein in synovial fluids from patients with various joint diseases. Arthritis Rheum.

[51] Vynios DH (2006). Autoantibodies against aggrecan in systemic rheumatic diseases. Biochimie.

[52] Perez-Carretero C (2020). Chronic lymphocytic leukemia patients with IGH translocations are characterized by a distinct genetic landscape with prognostic implications. Int J Cancer.

[53] Puente XS (2015). Non-coding recurrent mutations in chronic lymphocytic leukaemia. Nature.

[54] Zang H (2020). Circ-RNF111 contributes to paclitaxel resistance in breast cancer by elevating E2F3 expression via miR-140-5p. Thorac Cancer.

[55] Levin M (2006). Anti-interferon auto-antibodies in autoimmune polyendocrinopathy syndrome type 1. PLoS Med.

[56] Meager A (2006). Anti-interferon autoantibodies in autoimmune polyendocrinopathy syndrome type 1. PLoS Med.

[57] Meyer S (2016). AIRE-deficient patients harbor unique high-affinity disease-ameliorating autoantibodies. Cell.

[58] Walter JE (2015). Broad-spectrum antibodies against self-antigens and cytokines in RAG deficiency. J Clin Invest.

[59] Rosenberg JM (2018). Neutralizing anti-cytokine autoantibodies against interferon-α in immunodysregulation polyendocrinopathy enteropathy X-linked. Front Immunol.

[60] Steinman RM (2003). Tolerogenic dendritic cells. Annu Rev Immunol.

[61] Savan R (2009). Structural conservation of interferon gamma among vertebrates. Cytokine Growth Factor Rev.

[62] Wu UI (2017). Neutralizing antiinterferon-γ autoantibodies causing disseminated Mycobacterium avium complex infection in an HIV-infected patient on successful combination antiretroviral therapy. AIDS.

[63] Beyer M (2007). Combinatorial synthesis of peptide arrays onto a microchip. Science.

[64] Vazquez SE (2022). Autoantibody discovery across monogenic, acquired, and COVID-19-associated autoimmunity with scalable PhIP-seq. Elife.

