# Supplementary material for: Neutralizing IFN-γ autoantibodies are rare and pathogenic in HLA-DRB1*15:02 or 16:02 individuals
Source: J Clin Invest. 2024 Mar 12;134(8):e178263. doi: 10.1172/JCI178263 (PMC11014650; doi:10.1172/JCI178263)
Supplement: Supplemental data [file jci-134-178263-s157.pdf]

Supplemental Table 1

Patient	Sex	Follow	Age at onset of symptoms	Clinical phenotype (infections, microbiology, other diseases)	Neutralizing auto-Abs to cytokines	Other auto-Abs	Reference
P1	F	Dead	52 years	Disseminated infection by *M. fortuitum*<br>Recurrent disseminated infection by *M. avium*<br><br>Autoimmune thyroiditis		SLE ANA(+)<br>Anti-SSA(+)<br>ASMA (-)<br>AMA (-)<br>Anti-LKM1/anti-E2PDH/anti LC1/anti-SLA (-)	Ku CL et al. J Allergy Clin Immunol 2016, 137(3):954-8.
P2	F	Alive	50 years	Disseminated infection by *M. fortuitum*<br>Osteomyelitis by *M. intracellulare*<br>Recurrent thoracic varicella zoster	Anti-GM-CSF (+)	SLE ANA(-)<br>Anti-SSA(-)<br>ASMA (-)<br>AMA (-)<br>Anti-LKM1/anti-E2PDH/anti LC1/anti-SLA (-)	Valour F et al, Emerg Infect Dis 2016, 22(6):1124-1126<br><br>Ku CL et al. J Allergy Clin Immunol 2016, 137(3):954-8.
P3	F	Alive	44 years	Adenitis by *M. fortuitum*<br>Adenitis by *P. marfaneii*<br>Adenitis by *M. scrofulaceum*<br><br>Autoimmune thyroiditis, sclerodermia		SLE ANA(+)<br>Anti-SSA(-)<br>ASMA (-)<br>AMA (-)<br>Anti-LKM1/anti-E2PDH/anti LC1/anti-SLA (-)	Ku CL et al. J Allergy Clin Immunol 2016, 137(3):954-8.
P4	M	Alive	29 years	Disseminated infection by *M. fortuitum*		SLE ANA(+)<br>Anti-SSA(-)<br>ASMA (+)<br>AMA (-)<br>Anti-LKM1/anti-E2PDH/anti LC1/anti-SLA (-)	This paper
P5	M	Alive	52 years	Osteomyelitis by *Histoplasma capsulatum*<br>Disseminated infection by *M. avium*<br>*Herpes* Simplex ophthalmicus		SLE ANA(-)<br>Anti-SSA(-)<br>ASMA (-)<br>AMA (-)<br>Anti-LKM1/anti-E2PDH/anti LC1/anti-SLA (-)	This paper
P6	M	Alive	36 years	Spondylodiscitis L5-S1, paravertebral abscess by *M. avium*		SLE ANA(ND)<br>Anti-SSA(ND)<br>ASMA (ND)<br>AMA (ND)<br>Anti-LKM1/anti-E2PDH/anti LC1/anti-SLA (ND)	This paper
P7	M	Alive	53 years	Severe infection by *Legionella pneumonia*<br>Recurrent disseminated infection by *M. avium*			This paper

Abbreviations: ANA: antinuclear antibody, ASMA: anti-smooth muscle antibody; AMA: anti-mitochondria; ND not determined

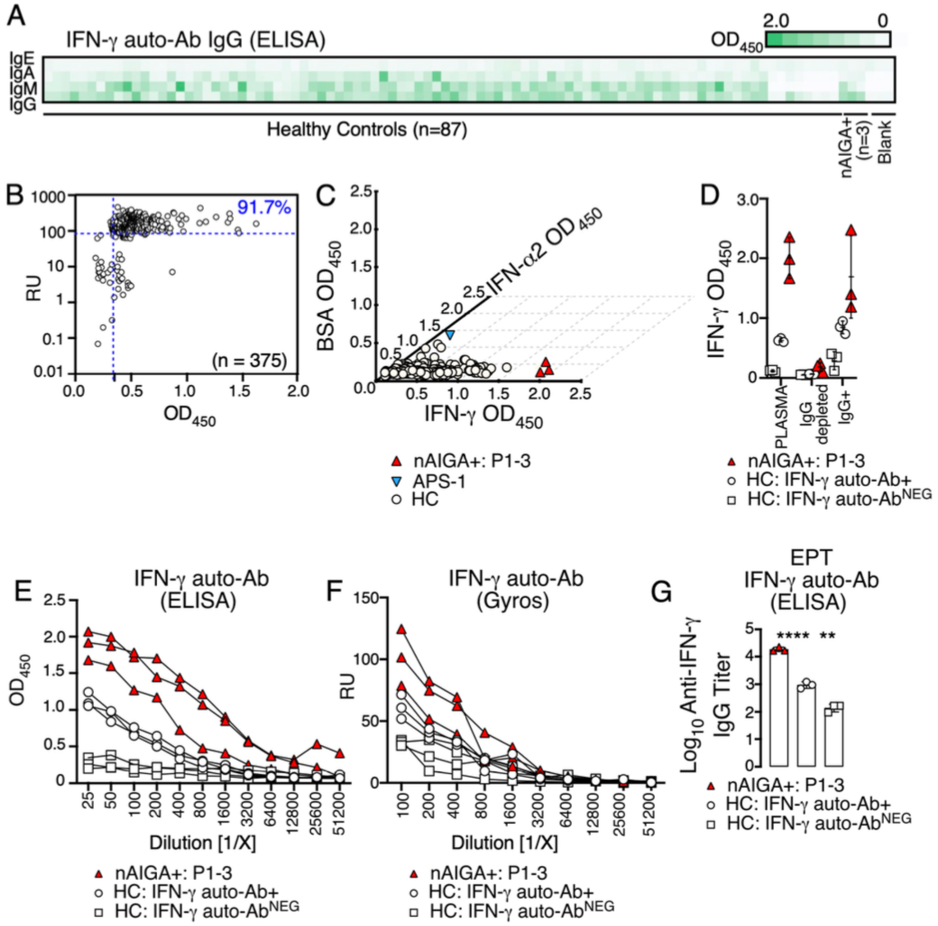

Supplemental Figure 1. related to Figure 1 IFN-γ auto-Abs are detected in the general population though not neutralizing.

(A) A heatmap displays IFN-γ auto-Abs (IgM, IgA, IgG) detectable in 87 healthy controls of the general population, ranging from lowest to highest optical densities (OD450).

(B) Correlation analysis between IFN-γ auto-Abs detection by Gyros and ELISA, with a subset of individuals enriched for Gyros-positive results. The positivity threshold is indicated by a blue dotted line.

(C) Correlation between IFN-γ auto-Abs detection and other antigens (IFN-α2 and BSA) among individuals of the general population, nAIGA patients, and APS-1 patients.

(D) IgG purification and IFN-γ auto-Abs detection by ELISA in whole plasma, IgG-depleted fraction, and IgG-purified fraction of patients with EM infections, healthy controls with and without detectable IFN-γ auto-Abs, and nAIGA patients.

(E) Titers of plasma from IFN-γ auto-Abs-positive healthy controls, IFN-γ auto-Abs-negative healthy controls, and patients with EM infections due to nAIGA, as determined by ELISA.

(F) Titers of plasma from IFN-γ auto-Abs-positive healthy controls, IFN-γ auto-Abs-negative healthy controls, and patients with EM infections due to nAIGA, as determined by Gyros.

(G) Endpoint titer determined by the Log10(lowest detectable dilution) from IFN-γ auto-Abs-positive healthy controls, IFN-γ auto-Abs-negative healthy controls, and patients with EM infections due to nAIGA.

Data represent two experiments (A, C-G), with each sample tested once for large-scale screening (B). Statistical significance was calculated using an unpaired student's T test, with significance denoted as $*p < 0.05$, $**p \leq 0.01$, $***p \leq 0.001$, $****p \leq 0.0001$, or unmarked for nonsignificance.

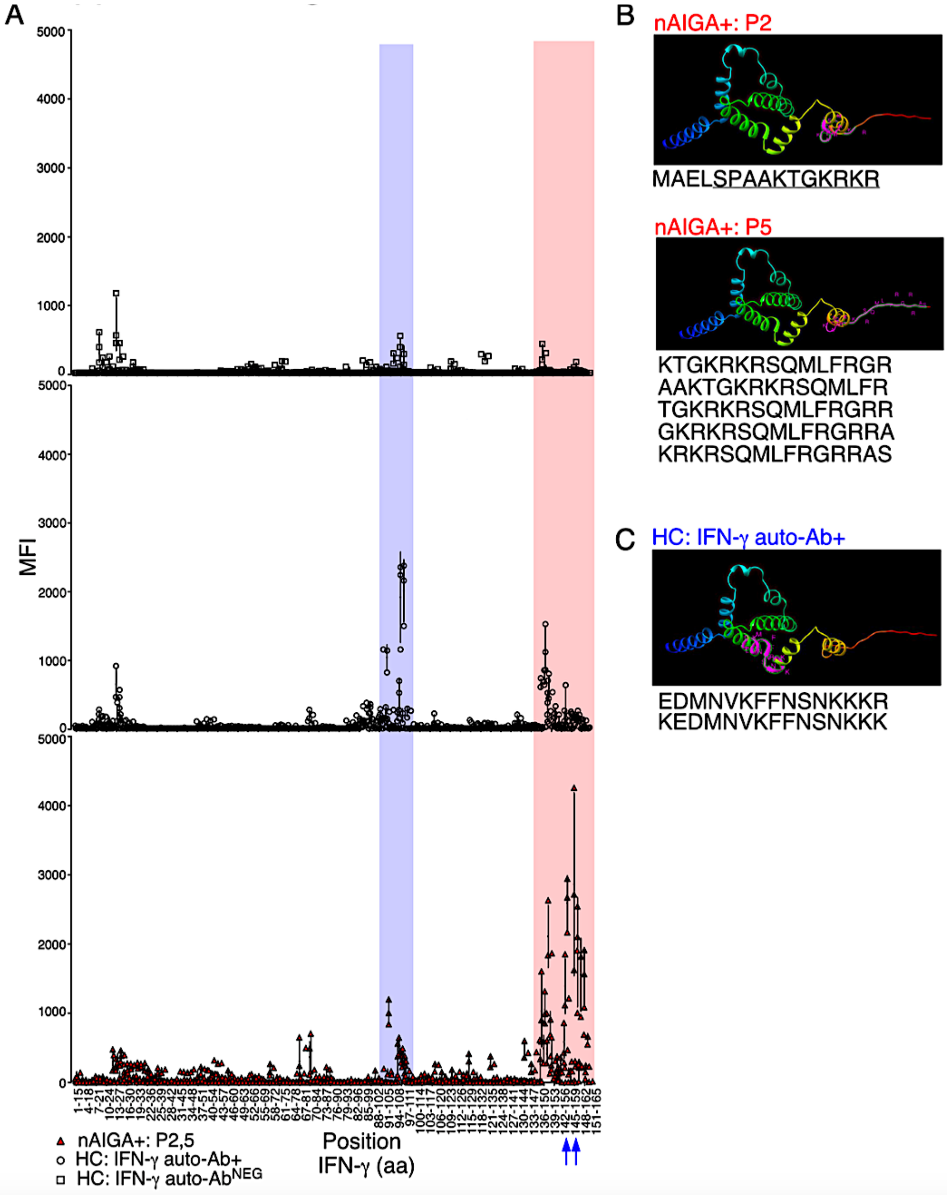

Supplemental Figure 2 related to Figure 2

IFN-γ auto-Abs of the general population are of low affinity and are functionally distinct.

(**A**) 2 individuals of the general population that were negative for detection against full length IFN-γ auto-Abs (white squares, top), 2 individuals of the general population that were positive for detection against full length IFN-γ auto-Abs (white circles, middle), and 2 nAIGA patients (red triangles, bottom) were assayed by a custom peptide microarray from PepperPrint in duplicate. Mean fluorescent intensities (MFI) were plotted with respect to amino acid position of IFN-γ. Regions of significant epitopes are highlighted in blue and red.

(**B**) Predicted structure of IFN-γ from AlphaFold protein structure database, epitopes as determined by (A, red) in magenta from patients harboring nAIGA.

(**C**) Predicted structure of IFN-γ from AlphaFold protein structure database, epitopes as determined by (A, blue) in magenta from a healthy control (HC) harboring IFN-γ auto-Abs.

Each sample was tested once. Triplicate is a technical replicate.

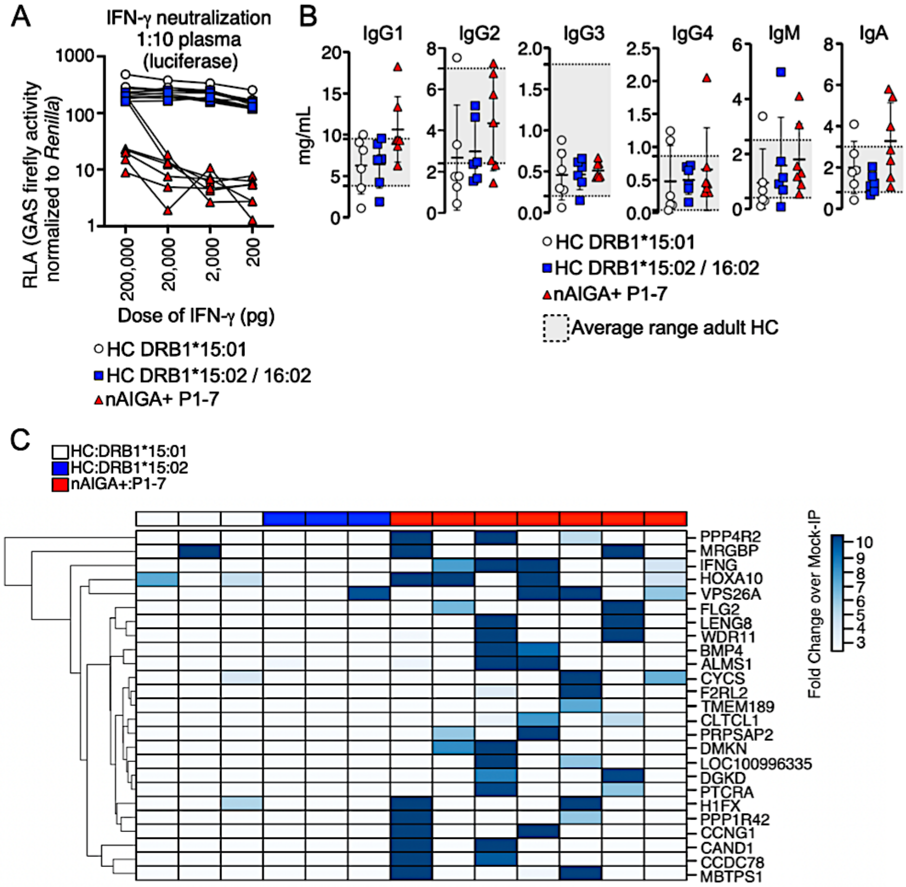

Supplemental Figure 3 related to Figure 4

nAIGA are rare and are not detected in those with autoimmune conditions.

(**A**) Results for the neutralization of IFN-γ (200 pg/ml-20 ng, final concentration) in the presence of plasma 1:10 from (7) patients with EM infections due to nAIGA (red triangles), (3) HLA-DRB1*15:02 and/or 16:02 carriers (blue squares), and (3) HLA-DRB1*15:01 carriers (white circles). Relative luciferase activity is shown (GAS dual luciferase activity, with normalization against *Renilla* luciferase activity) after stimulation with IFN-γ (200 pg/ml-20 ng) in the presence of plasma 1:10. RLA, relative luciferase activity.

(**B**) Total Ig assessed in the serum from (7) patients with EM infections due to nAIGA (red triangles), (3) HLA-DRB1*15:02 and/or 16:02 carriers (blue squares), and (3) HLA-DRB1*15:01 carriers (white circles) by Legendplex.

(**C**) Heatmap of top specific auto-Abs in the serum from (7) patients with EM infections due to nAIGA (red triangles), (3) HLA-DRB1*15:02 and/or 16:02 carriers (blue squares), and (3) HLA-DRB1*15:01 carriers (white circles) as assessed by PhIP-seq.

Data are representative of 2 experiments (**A**, **B**). For PhIP-seq (**C**) each sample was tested once.
